# From preoperative to postoperative: gender differences in elective ventral hernia repair

**DOI:** 10.1007/s10029-026-03678-8

**Published:** 2026-04-17

**Authors:** William Head, Courtney M. Collins, Ashley Aldridge, Li-Ching Huang, Marisa Blackman, Courtney E. Collins

**Affiliations:** 1 Center for Abdominal Core Health, Department of Surgery, The Ohio State University Wexner Medical Center, 410 W. 10Th St., Columbus, OH 43210 USA; 2 Department of Biostatistics, Vanderbilt University Medical Center, Nashville, USA

**Keywords:** Ventral Hernia, Hernia Repair, Gender

## Abstract

**Purpose:**

Gender represents an important social construct, yet its impact on hernia care is poorly understood. This study aims to evaluate gender-based differences across the continuum of elective ventral hernia management.

**Methods:**

A retrospective cohort study was conducted for adults undergoing elective ventral hernia repair with mesh in the Abdominal Core Health Quality Collaborative registry. Patients were categorized as men or women. Variables analyzed included demographics, comorbidities, hernia characteristics, operative decision-making, postoperative outcomes, and patient-reported outcomes (PROs). Multivariable regression modeling evaluated associations between gender and postoperative outcomes.

**Results:**

27,046 patients were evaluated (53% men, 47% women). Women less often had private insurance (48% vs 56%, p < 0.001) and presented more frequently with larger (width 5 cm vs 4 cm; length 8 cm vs 4 cm, p < 0.001) and recurrent hernias (31% vs 24%, p < 0.001). Operative approach did not differ, yet women more often underwent lengthier repairs (> 2 h 53% vs 40%, p < 0.001) and myofascial release (44% vs 34%, p < 0.001). Women had worse adjusted length of stay (effect 0.219 days, p < 0.001), surgical site infection within 30 days (OR 1.310, p < 0.001), and surgical site infection/occurrence interventions within 30 days (OR 1.144, p = 0.047). They also reported lower quality of life and pain interference at baseline with greater gains across two years (p < 0.001).

**Conclusion:**

Women presented with more advanced hernia disease requiring more complex reconstruction with worse early morbidity. Despite this, women achieved greater gains in PRO scores relative to men. These findings highlight the need for earlier recognition and targeted optimization to ensure equitable management patterns of ventral hernias.

**Supplementary Information:**

The online version contains supplementary material available at 10.1007/s10029-026-03678-8.

## Introduction

Ventral hernias are among the most commonly addressed surgical problems with more than 600,000 repaired annually in the United States [1]. Ventral hernia repair (VHR) outcomes are strongly influenced by a complex interplay of surgical technique, hernia characteristics, and patient factors. Among these, patient factors have become an important area of study, since they provide the opportunity for both risk stratification and preoperative optimization. Factors such as age, body mass index (BMI), comorbidities (diabetes, chronic obstructive pulmonary disease [COPD], immunosuppression), smoking status, and American Society of Anesthesiologists (ASA) class, have been consistently linked with increased complications and recurrence [2, 3]. Although these variables are well-established in the literature, one fundamental patient characteristic remains poorly understood – gender.

Gender and sex are distinct yet often inter-related concepts that influence surgical outcomes. “Sex” traditionally refers to the biologic and physiologic traits that distinguish male versus female; whereas “gender” refers to a range of social and cultural identities that can include but are not limited to man and woman. Sex-based differences have previously been evaluated in the hernia population with noted relevance to abdominal wall mechanics. Females tend to have greater subcutaneous abdominal fat volume while males have greater intra-abdominal visceral fat [4]. Females on average also have a higher hernia-volume to intra-abdominal volume ratio on computerized tomography imaging [4]. Female fascia has also been described as having increased stiffness at advanced ages [5]. Gender-based differences have also been highlighted across the healthcare system with notable disparities in access, quality, and outcomes. Women often face greater barriers to obtaining care and are more likely to report delays in care as well as decision-making bias [6, 7]. Women consistently present with poorer baseline quality of life scores though, yet their degree of improvement after surgery is often similar [8–10]. Ultimately, these disparities have been linked to higher overall mortality and greater loss of healthy life years for women compared to men [11].

A growing body of literature is identifying significant gender-based disparities in outcomes across multiple surgical specialties. Currently, there exists a knowledge gap with respect to gender and the ventral hernia population. The aim of this study is to evaluate gender-based differences across the spectrum of elective ventral hernia management – from preoperative presentation to intraoperative decision-making and, finally, postoperative outcomes. We hypothesize that women undergoing elective VHR present with a worse preoperative risk profile, undergo comparable operative management, and experience worse outcomes relative to men. By evaluating these trends, this study seeks to clarify the relevance of gender to hernia care and identify opportunities for tailored management strategies.

## Materials & methods

This research was determined by the authors’ Institutional Review Board to fall into one or more categories of exemption from review as established by federal regulations.

### Study population

The study population was derived from the Abdominal Core Health Quality Collaborative (ACHQC) [12]. The ACHQC is an international 501(c)(3) non-profit Center for Medicare and Medicaid Services (CMS) Qualified Clinical Data Registry that focuses on long-term follow-up data and patient-reported outcomes related to abdominal core and hernia surgical repairs. Informed consent was waived given the use of de-identified registry data. The study incorporated all documented VHR cases between January 2016 and December 2023. Case inclusion criteria were adults (≥ 18 years old), wound classes I-III, elective case status, and mesh implantation. Exclusion criteria were parastomal hernia repair, missing gender/race/smoking/diabetes status, and fewer than 30-days of postoperative follow-up. Patients were stratified into binary gender groups: men and women.

### Study design

A retrospective cohort study was conducted using the ACHQC registry. Data was evaluated across the preoperative, operative, and postoperative timeframes with focus on binary gender-based comparisons. Queried preoperative variables of interest included demographics (gender, age, race, and insurance status), baseline patient information (functional status, ASA class, BMI, diabetes mellitus status, and current smoking status), and hernia characteristics (transverse width, craniocaudal length, Modified Ventral Hernia Working Group grade, initial vs recurrent, number of prior repairs, and indication). Indication for hernia repair reasons included the following: bowel obstruction, fistula, infected mesh, enlarging/interfering with activities, pain, and asymptomatic. Operative variables of interest included approach (open, laparoscopic, robotic, minimally invasive converted to open), operative time, prophylactic antibiotic administration, any concomitant procedures, wound status, mesh type and location, myofascial release, subcutaneous flap creation, drain use, and intraoperative complication(s). Postoperative variables of interest included hospital length of stay (LOS), pertinent outcomes within 30 days (readmission, reoperation, surgical site infection [SSI], surgical site occurrences [SSO], SSI/SSO requiring intervention, pain requiring intervention), recurrence (at 1-year, 2-years, and 3-years), mortality within 44 days, and patient-reported outcomes. Patient-reported outcomes included the Hernia-Related Quality of Life Survey (HerQLes) scores and the Patient-Reported Outcomes Measurement Information System (PROMIS) Pain T scores at baseline, 30 days, 1 year, and 2 years [13, 14].

### Statistical analysis

Statistical expertise was available to the authors. Continuous variables were reported as median values with interquartile ranges and compared using the Wilcoxon rank-sum test. Categorical variables were reported as frequencies with percentages and compared using the Pearson chi-square test. Multivariable linear and logistic regression modeling was utilized to compare postoperative outcomes across gender, reporting effect sizes and odds ratios with 95% confidence intervals. Due to the small missing rate (< 3%), only completed cases were included in the regression analyses. The following covariates were selected a priori based on clinical relevance: hernia width (< 4 cm vs ≥ 4–10 cm vs ≥ 10 cm), BMI (< 30 kg/m^2^ vs ≥ 30–35 kg/m^2^ vs ≥ 35–40 kg/m^2^ vs ≥ 40 kg/m^2^), smoking status (active vs inactive), diabetes, ASA class (1 vs 2 vs 3 vs 4), immunosuppressant use, COPD, and functional status (independent vs partially/totally dependent). The postoperative outcomes evaluated by the models were LOS, SSI, SSO, SSI/SSO requiring intervention, 30-day readmission, 30-day reoperation, 1-year recurrence, 2-year recurrence, and 3-year recurrence. All analyses were conducted using R version 4.3 [15]. Statistical significance was defined using a p-value of < 0.05. Figures were generated using Python with Matplotlib version 3.9.0 [16].

## Results

### Preoperative evaluation

A total of 27,046 patients met the relevant inclusion/exclusion criteria with a near equal gender distribution (14,259 men [53%] and 12,787 women [47%]). The median age was similar (men 58 years vs women 57 years; p < 0.001). Women more frequently identified as a racial minority (21% vs 13%; p < 0.001) and less often had private insurance (48% vs 56%; p < 0.001). No clinically significant difference was identified with respect to functional status (independent: men 97% vs women 98%; p < 0.001) nor ASA class (2: men 45% vs women 40%; 3: men 46% vs women 51%; p < 0.001). Despite statistical significance, BMI and rates of diabetes were essentially comparable between groups (32 kg/m^2^ vs 31 kg/m^2^; p < 0.001) (19% vs 17%; p < 0.001). There was no statistically significant difference with respect to current smoking status (men 10% vs women 10%; p = 0.2). See Table 1 for further preoperative patient details.

**Table 1:** Preoperative Patient Characteristics by Gender

	Overall	Men	Women	P-value
N	27,046	14,259 (53%)	12,787 (47%)	
Age (years)	58 (47, 67)	58 (49, 67)	57 (46, 67)	< 0.001
Race (minority)	4569 (17%)	1886 (13%)	2683 (21%)	< 0.001
Primary insurance				< 0.001
- Private	14,054 (52%)	7964 (56%)	6090 (48%)	
- Medicare	8169 (30%)	4019 (28%)	4150 (32%)	
- Medicaid	2228 (8%)	803 (6%)	1425 (11%)	
- Other/unknown	2594 (10%)	1472 (10%)	1122 (9%)	
Functional Status				< 0.001
- Independent	26,439 (98%)	14,007 (98%)	12,432 (97%)	
- Partially Dependent	358 (1%)	144 (1%)	214 (2%)	
- Totally Dependent	30 (0%)	10 (0%)	20 (0%)	
- Unknown	212 (1%)	93 (1%)	119 (1%)	
ASA (class)				< 0.001
- 1	1827 (7%)	988 (7%)	839 (7%)	
- 2	11,517 (43%)	6346 (45%)	5171 (40%)	
- 3	13,012 (48%)	6524 (46%)	6488 (51%)	
- 4	515 (2%)	285 (2%)	230 (2%)	
- 5	0 (0%)	0 (0%)	0 (0%)	
- None Assigned	166 (1%)	109 (1%)	57 (0%)	
Body Mass Index (kg/m^2^)	32 (28, 36)	31 (28, 35)	32 (28, 37)	< 0.001
Diabetes Mellitus	4803 (18%)	2379 (17%)	2424 (19%)	< 0.001
Current Smoker	2647 (10%)	1428 (10%)	1219 (10%)	0.2

ASA: American Society of Anesthesiologists Physical Status Classification System. Median and frequency reported where appropriate with interquartile range and percentiles in parentheses, respectively

The overall median hernia transverse width and craniocaudal length were 4 cm (IQR 2–9) and 6 cm (IQR 2–15), respectively. Women had larger hernias with respect to both the median width (5 cm vs 4 cm; p < 0.001) and length (8 cm vs 4 cm; p < 0.001). Women also more frequently had grade 3 hernias (8% vs 5%; p < 0.001) as well as recurrent hernia repairs (31% vs 24%; p < 0.001). Indications for repair varied across gender with women more frequently undergoing repair for bowel obstruction (6% vs 3%; p < 0.001) and pain (89% vs 83%; p < 0.01). See Table 2 for further preoperative hernia details.

**Table 2:** Preoperative Hernia Characteristics by Gender

	Overall	Men	Women	P-value
Width (cm)	4 (2, 9)	4 (2, 8)	5 (3, 10)	< 0.001
Length (cm)	6 (2, 15)	4 (2, 14)	8 (3, 15)	< 0.001
Grade				< 0.001
- 1	7713 (29%)	4146 (29%)	3567 (28%)	
- 2	17,653 (65%)	9398 (66%)	8255 (65%)	
- 3	1680 (6%)	715 (5%)	965 (8%)	
Recurrent	7278 (27%)	3356 (24%)	3922 (31%)	< 0.001
Number of prior hernia repairs	1 (1, 2)	1 (1, 2)	1 (1, 2)	< 0.001
Indication				
- Bowel obstruction	1175 (4%)	472 (3%)	703 (6%)	< 0.001
- Fistula	74 (0%)	40 (0%)	34 (0%)	0.8
- Infected mesh	86 (0%)	40 (0%)	46 (0%)	0.3
- Enlarging/interfering with activities	17,769 (66%)	9388 (66%)	8381 (66%)	0.4
- Pain	22,999 (86%)	11,732 (83%)	11,267 (89%)	< 0.001
- Asymptomatic	641 (2%)	424 (3%)	217 (2%)	< 0.001

Frequency reported with percentiles in parentheses

### Operative management

Although statistically significant, the distribution of operative approach and wound class did not differ clinically (open: men 56% vs women 52%, laparoscopic: men 11% vs women 12%, robotic: men 32% vs women 35%, MIS conversion to open: men 1% vs women 1%; p < 0.001) (clean: men 95% vs women 92%, clean-contaminated: men 4% vs women 6%, contaminated: men 1% vs women 2%; < 0.001). Women more often had operative times > 2 h (53% vs 40%; p < 0.001). Mesh type did not differ from a clinically relevant perspective with the majority being permanent synthetic. Women did more frequently have retromuscular sublay placement (49% vs 37%; p < 0.001) while men more frequently had preperitoneal sublay placement (39% vs 31%; p < 0.001). Women also had increased rates of myofascial release (44% vs 34%; p < 0.001), subcutaneous flap creation (18% vs 14%; p < 0.001) and drain placement (42% vs 31%; p < 0.001). Women also had increased rates of concomitant procedures (17% vs 11%; p < 0.001) with the most notable categories being Soft Tissue/Plastics and Obstetric/Gynecologic. No clinically significant differences were identified regarding intraoperative complication(s) (men 2% vs women 2%; p = 0.01). See Table 3 for further operative details.

**Table 3:** Operative Details by Gender

	Overall	Men	Women	P-value
Approach				< 0.001
- Open	14,615 (54%)	8008 (56%)	6607 (52%)	
- Laparoscopic	3101 (11%)	1588 (11%)	1513 (12%)	
- Robotic	9053 (33%)	4558 (32%)	4495 (35%)	
- MIS convert to open	276 (1%)	105 (1%)	171 (1%)	
Operative time > 2 h	12,532 (46%)	5713 (40%)	6819 (53%)	< 0.001
Prophylactic antibiotic	26,372 (98%)	13,884 (97%)	12,488 (98%)	0.1
Concomitant procedure	3667 (14%)	1499 (11%)	2168 (17%)	< 0.001
Wound status				< 0.001
- Clean	25,366 (94%)	13,544 (95%)	11,822 (92%)	
- Clean-contaminated	1313 (5%)	542 (4%)	771 (6%)	
- Contaminated	367 (1%)	173 (1%)	194 (2%)	
Primary mesh type				< 0.001
- Biologic	539 (2%)	255 (2%)	284 (2%)	
- Permanent synthetic	25,707 (95%)	13,646 (96%)	12,061 (94%)	
- Resorbable synthetic	783 (3%)	349 (2%)	434 (3%)	
- Other/unknown	14 (0%)	9 (0%)	5 (0%)	
Primary mesh location				< 0.001
- Onlay	1553 (6%)	700 (5%)	853(7%)	
- Inlay	592 (2%)	272 (2%)	320(3%)	
- Sublay	24,900 (92%)	13,287 (93%)	11,613 (91%)	
Sublay Position				
- Retromuscular	10,624 (43%)	4967 (37%)	5657 (49%)	< 0.001
- Preperitoneal	9950 (36%)	5207 (39%)	3643 (31%)	< 0.001
- Intraperitoneal	8063 (32%)	4353 (33%)	3710 (32%)	0.2
Myofascial release	10,489 (39%)	4858 (34%)	5631 (44%)	< 0.001
Subcutaneous flaps	4223 (16%)	1973 (14%)	2250 (18%)	< 0.001
Drains used	9713 (36%)	4369 (31%)	5344 (42%)	< 0.001
Intraoperative complication(s)	554 (2%)	263 (2%)	291 (2%)	0.01

MIS: Minimally invasive surgery (laparoscopic or robotic). Frequency reported with percentiles in parentheses

### Postoperative outcomes

The unadjusted analysis showed that the median LOS was longer for women (1 day [IQR 0–4] vs 0 days [IQR 0–2]; p < 0.001) and more women were admitted for five or more days (18% vs 13%; p < 0.001). Women had slightly increased rates of 30-day readmission (4% vs 3%; p < 0.001), SSIs (3% vs 2%; p < 0.001), and SSOs (10% vs 9%; p < 0.001). The associated SSI/SSO procedural intervention rate within 30 days was also slightly higher for women (4% vs 3%; p < 0.001) as well as pain requiring intervention (8% vs 2%; p < 0.001). No significant differences were noted with respect to 1-year recurrence or mortality. Women had slightly higher rates of recurrence at 2-years (19% vs 17%; p = 0.01) and 3-years (21% vs 18%; p = 0.01). See Table 4 for further postoperative outcome details.

**Table 4:** Postoperative Outcomes by Gender

	Overall	Men	Women	P-value
Length of stay (days)	0 (0, 3)	0 (0, 2)	1 (0, 4)	< 0.001
≥ 5-day admission	4152 (15%)	1876 (13%)	2276 (18%)	< 0.001
Readmission within 30 days	962 (4%)	456 (3%)	506 (4%)	< 0.001
Readmission reason				
- Wound	319 (38%)	124 (31%)	195 (44%)	< 0.001
- Bleeding	70 (8%)	41 (10%)	29 (7%)	0.05
- Thrombotic	27 (3%)	8 (2%)	19 (4%)	0.06
- Gastrointestinal	279 (33%)	158 (40%)	121 (27%)	< 0.001
Reoperation within 30 days	330 (1%)	162 (1%)	168 (1%)	0.2
SSI within 30 days	733 (3%)	306 (2%)	427 (3%)	< 0.001
SSO within 30 days	2500 (9%)	1213 (9%)	1287 (10%)	< 0.001
SSI/SSO procedural intervention ≤ 30 days	1002 (4%)	443 (3%)	559 (4%)	< 0.001
Pain requiring intervention ≤ 30 days	80 (5%)	19 (2%)	61 (8%)	< 0.001
Recurrence				
- 1 year	723 (12%)	351 (12%)	372 (13%)	0.2
- 2 years	776 (18%)	370 (17%)	406 (19%)	0.01
- 3 years	591 (20%)	274 (18%)	317 (21%)	0.01
Mortality within 44 days	39 (0%)	23 (0%)	16 (0%)	0.4

SSI: Surgical site infection. SSO: Surgical site occurrence. Frequency reported with percentiles in parentheses

**Fig. 1:**
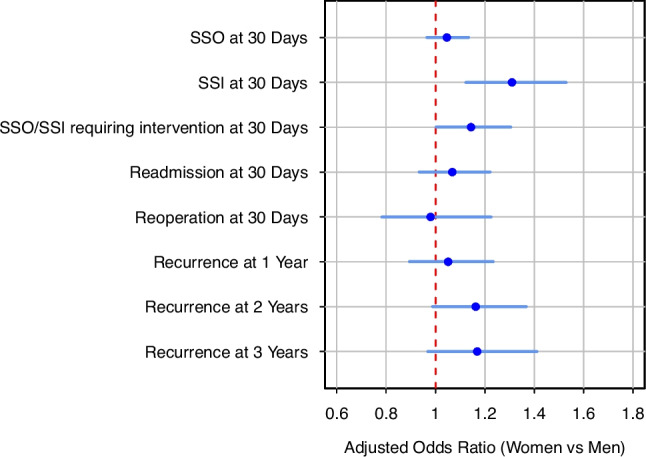
Multivariable Regression Modeling of Gender Associations with Postoperative Outcomes

Multivariable linear and logistic regression modeling identified several differences across gender. The analysis confirmed that women had longer mean adjusted hospital LOS (effect 0.219 days, 95% CI 0.135–0.303; p < 0.001). They also had higher odds of SSI within 30 days (OR 1.310, 95% CI 1.123–1.529; p < 0.001) and SSI/SSO requiring a procedural intervention within 30 days (OR 1.144, 95% CI 1.002–1.305; p = 0.047). The models found no evidence of gender-based differences with respect to SSO within 30 days, readmission and/or reoperation within 30 days, and recurrence at 1 and/or 2 and/or 3 years. These models incorporated clinically relevant covariates related to comorbidities and hernia characteristics, allowing adjustment for factors that may differ by gender and influence postoperative outcomes. See Fig. 1 for a forest plot of the adjusted odds ratios for women versus men from the multivariable logistic regression models. See Supplemental Table 1 for the full model outputs.

**Fig. 2:**
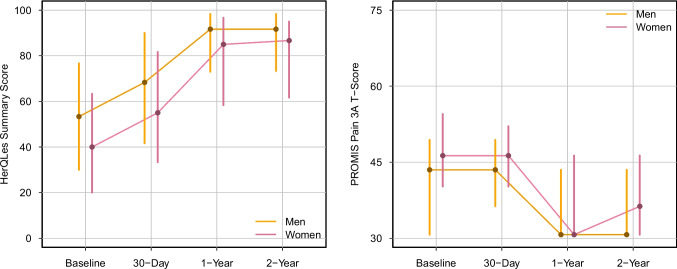
Patient-Reported Outcome Absolute Scores

**Fig. 3:**
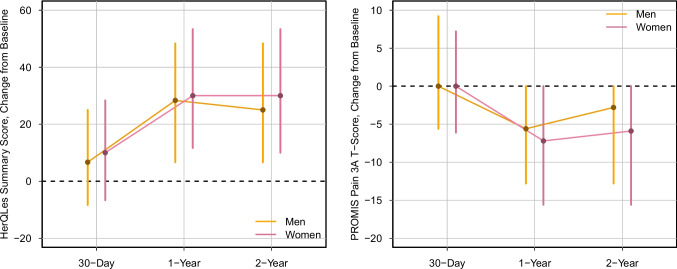
Patient-Reported Outcome Score Changes from Baseline

Patient-reported outcomes differed by gender across all follow-up times for both HerQLes and PROMIS Pain. Figures 2 and 3 display absolute scores and change-from-baseline trajectories, respectively. Women reported lower quality of life at baseline (median HerQLes score 40 vs 53; p < 0.001) and postoperatively at 30 days (55 vs 68; p < 0.001), 1 year (85 vs 92; p < 0.001), and 2 years (87 vs 92; p < 0.001). Similarly, women reported higher pain interference at baseline (median PROMIS score 46 vs 44; p < 0.001) and postoperatively at 30 days (46 vs 44; p < 0.001), 1 year (31 [IQR 31–46] vs 31 [IQR 31–44]; p < 0.001), and 2 years (36 vs 31; p < 0.001). Change-from-baseline analysis showed clinically meaningful improvements regardless of gender across 2 years. However, women experienced significantly greater relative gains at each timepoint for both HerQLes and PROMIS Pain. See Supplementary Tables 2 and 3 for further patient-reported outcome details.

## Discussion

This retrospective cohort evaluation of the ACHQC data registry provides an evaluation of gender-based differences across the spectrum of elective ventral hernia management. Preoperatively, women presented as a more diverse patient population with larger hernias and a clinically similar risk profile when compared to men. Intraoperatively, women more frequently required lengthier operations with more complex reconstructions. Postoperatively, women experienced longer lengths of stay with greater early wound morbidity. Women also had worse baseline reported quality of life (QOL) and pain interference due to their hernias but experienced greater postoperative improvements in QOL and pain relative to men. This study and its findings identify several key themes that help clarify the relevance of gender in hernia care with implications for tailored management strategies moving forward.

Women presented with broadly similar medical risk profiles to men, including comparable age, functional status, ASA class distribution, BMI, diabetes prevalence, and smoking status. However, a primary difference was the social factors. Women were disproportionately represented among minority racial groups, being ~ 60% more likely to identify as a minority, and were ~ 15% less likely to have private insurance. These markers of social vulnerability have been validated in the literature and can result in patients having less access to timely care, delayed referrals, presentation with advanced disease, longer hospital stays, and increased morbidity and mortality [14–21]. For women who belong to racial minority groups, these disadvantages may be compounded by intersecting sociodemographic factors that place them at heightened risk. This study’s findings ultimately reflect these generalized disparities as women presented with more advanced hernia disease. Their defects were on average 25% wider and 100% longer, and they were ~ 30% more likely to present with a recurrent hernia. Similar hernia characteristic trends with respect to size and recurrence were also recently identified in a retrospective review of the Michigan Surgical Quality Collaborative Hernia Registry [22]. These preoperative differences carry important clinical implications and ultimately may be reflected in the gender-based disparities noted for operative decision-making and postoperative outcomes.

Operative complexity varied by gender in ways that potentially reflect underlying disease severity rather than surgeon preference. Although the initial operative approach and mesh type did not differ considerably, women were ~ 29% more likely to undergo myofascial release, ~ 29% more likely to have subcutaneous flaps created, and ~ 36% more likely to receive drains. These techniques are commonly reserved for larger and recurrent defects, which women had in this study. Prior work has identified higher rates of wound complications, such as infection, seroma, and necrosis, with these interventions that can meaningfully delay short-term recovery and patient wellbeing [23–27]. Women were also 33% more likely to undergo procedures exceeding two hours. Several studies have also shown a direct relationship between hernia size and recurrent repairs with operative time [28, 29]. While women in this study more often underwent concomitant procedures, we suspect the primary driver to be worse hernia phenotype at repair. The intraoperative complications did not differ clinically, suggesting that surgeons can achieve technical safety despite these increased reconstruction demands. A major aspect that is often outside the scope of the surgeon’s direct control though is postoperative outcomes.

Women had relatively worse postoperative outcomes in several important categories. In unadjusted analysis, they had longer length of stay as well as higher rates of 30-day readmission, SSIs, SSOs, SSI/SSO requiring procedural intervention, pain requiring intervention, and 2-year and 3-year recurrence. Adjusted analysis with multivariable regression modeling similarly identified longer length of stay and increased rates of 30-day SSI and SSI/SSO requiring procedural intervention. While the modeling controlled for key factors – hernia width, BMI, smoking status, diabetes, ASA class, immunosuppressant use, COPD, and functional status, we suspect that these factors may not be entirely independent of gender. Gender represents a continuum of social identities that may be inter-related with some of these variables and, therefore, adjusting for them may partially adjust away pathways through which gender influences outcomes. Prior studies have highlighted gender as a complex social determinant that is intertwined with other social identities and surgical risk factors [30, 31]. Gender is inherently related to sex as well, yet the influence of gender- and sex-based factors on postoperative outcomes is not clearly understood. Future studies are required to disentangle the relative contributions of each for the ventral hernia population.

Nevertheless, women reported worse baseline quality of life and pain interference in this study and subsequently experienced greater improvements for both after repair when compared to men. These findings have been reflected in other surgical populations as well where women report worse baseline functional disability, mood impairment, and perceptions of general health [8, 32, 33]. The gap in PROs has also been shown to dissipate over time across other surgical populations with women reporting similar benefit [9, 33]. Overall, women had somewhat worse postoperative outcomes but experienced relatively greater improvements in their reported wellbeing after repair. Tying in the pre- and intraoperative findings, we propose that women experience inequity across the continuum of surgical care for ventral hernias. These patterns highlight a need for earlier recognition of hernias and referrals for women to limit delayed presentation witnessed in this study. A more equitable approach may improve postoperative outcomes while maintaining the significant quality of life gains women achieve after hernia repair.

This study has several limitations that deserve consideration. First, its retrospective observational design limits causal inference. Second, the use of a voluntary, provider-driven data registry may introduce selection and information bias such that non-representative samples and misclassification of variables exist. ACHQC data largely originates from specialized, high-volume centers such that external generalizability may be limited. Third, although missing data was minimal, incomplete follow-up and underreporting may influence findings with respect to postoperative objective and patient-reported outcomes. Finally, gender was evaluated as a binary variable. In reality, gender includes a continuum of interacting identities that may not align strictly with “woman” or “man” for all patients. Future studies should incorporate a more gender-diverse cohort to further appreciate the impact of these identities on ventral hernia management.

## Conclusion

In this retrospective evaluation of 27,046 patients with ventral hernias, gender was associated with several key differences in the pre-, intra-, and postoperative stages of surgical care. Women presented with similar risk profiles but larger hernias that required more complex reconstruction and increased early morbidity. Women also had worse baseline quality of life and pain interference due to their hernias with relatively increased improvements in the long-term. Long-term recurrence did not differ notably after adjusted analysis. These findings highlight gender-based themes in hernia care that deserve attention when counseling patients, allocating resources, and designing optimization strategies. Future studies are needed to determine whether the observed disparities are due to differences in presentation, access to care, or current treatment pathways. By doing so, targeted strategies may be developed to ensure all genders receive equitable and effective ventral hernia care.

## Supplementary Information

Below is the link to the electronic supplementary material.

Supplementary file1 (DOCX 18 KB)

## Data Availability

The data utilized for this study was provided by the Abdominal Core Health Quality Collaborative (ACHQC) under a data use agreement and is not publicly available. De-identified data may be made available from the ACHQC upon reasonable request and approval by the ACHQC Data Use and Publications Committee.
