# Supplementary material for: From preoperative to postoperative: gender differences in elective ventral hernia repair
Source: Hernia. 2026 Apr 17;30(1):165. doi: 10.1007/s10029-026-03678-8 (PMC13090267; doi:10.1007/s10029-026-03678-8)
Supplement: Supplementary file 1 — Supplementary file1 (DOCX 18 KB) [file 10029_2026_3678_MOESM1_ESM.docx]

**SUPPLEMENTARY APPENDIX**

## **Supplementary Table 1: Multivariable Linear and Logistic Regression Models for Postoperative Outcomes and Gender**

| **Outcome** | **Model type** | **Effect** | **95% CI** | **p-value** |
| --- | --- | --- | --- | --- |
| Length of stay (days) | Linear | β = 0.219 | 0.135–0.303 | <0.001 |
| SSI within 30 days | Logistic | OR = 1.310 | 1.123–1.529 | <0.001 |
| SSO within 30 days | Logistic | OR = 1.046 | 0.965–1.134 | 0.27 |
| SSO/SSI requiring intervention within 30 days | Logistic | OR = 1.144 | 1.002–1.305 | 0.047 |
| Readmission within 30 days | Logistic | OR = 1.068 | 0.934–1.221 | 0.334 |
| Reoperation within 30 days | Logistic | OR = 0.980 | 0.783–1.225 | 0.857 |
| 1-year recurrence | Logistic | OR = 1.051 | 0.894–1.234 | 0.549 |
| 2-year recurrence | Logistic | OR = 1.163 | 0.988–1.368 | 0.069 |
| 3-year recurrence | Logistic | OR = 1.169 | 0.969–1.411 | 0.104 |

SSO: surgical site occurrence; SSI: surgical site infection; β: effect size; OR: Odds ratio. Models adjusted for the following covariates: hernia width, BMI, smoking status, diabetes, ASA class, immunosuppressant use, COPD, and functional status.

## **Supplementary Table 2: Patient Reported Outcomes for HerQLes by Gender**

|  | **N** | **Overall** | **Men** | **Women** | **P-value** |
| --- | --- | --- | --- | --- | --- |
| Score at Baseline | 10762 | 47 (25, 72) | 53 (30, 77) | 40 (20, 63) | <0.001 |
| Score at 30 Day | 12310 | 62 (38, 85) | 68 (42, 90) | 55 (33, 82) | <0.001 |
| Score at 1 Year | 4758 | 88 (65, 97) | 92 (73, 98) | 85 (58, 97) | <0.001 |
| Score at 2 Year | 3908 | 90 (68, 97) | 92 (73, 98) | 87 (62, 95) | <0.001 |
| Score change at 30 Day | 8168 | 8 (-7, 27) | 7 (-8, 25) | 10 (-7, 28) | <0.001 |
| Score change at 1 Year | 2964 | 30 (8, 52) | 28 (7, 48) | 30 (12, 53) | 0.001 |
| Score change at 2 Years | 2215 | 28 (8, 50) | 25 (7, 48) | 30 (10, 53) | <0.001 |

## HerQLes: Hernia-Related Quality of Life Survey. Median reported with interquartile range in parentheses. Score changes are relative to baseline score.

## **Supplementary Table 3: Patient Reported Outcomes for PROMIS Pain by Gender**

|  | **N** | **Overall** | **Men** | **Women** | **P-value** |
| --- | --- | --- | --- | --- | --- |
| Score at Baseline | 10748 | 46 (36, 52) | 44 (31, 49) | 46 (40, 54) | <0.001 |
| Score at 30 Day | 12298 | 46 (40, 52) | 44 (36, 49) | 46 (40, 52) | <0.001 |
| Score at 1 Year | 4748 | 31 (31, 44) | 31 (31, 44) | 31 (31, 46) | <0.001 |
| Score at 2 Year | 3879 | 31 (31, 46) | 31 (31, 44) | 36 (31, 46) | <0.001 |
| Score change at 30 Day | 8144 | 0 (-6, 8) | 0 (-6, 9) | 0 (-6, 7) | <0.001 |
| Score change at 1 Year | 2956 | -6 (-14, 0) | -6 (-13, 0) | -7 (-16, 0) | <0.001 |
| Score change at 2 Years | 2200 | -6 (-13, 0) | -3 (-13, 0) | -6 (-16, 0) | <0.001 |

PROMIS: **Patient-Reported Outcomes Measurement Information System**. Median reported with interquartile range in parentheses. Score changes are relative to baseline score.
